# Cardiac Autonomic Modulation and Response to Sub-Maximal Exercise in Chilean Hypertensive Miners

**DOI:** 10.3389/fphys.2022.846891

**Published:** 2022-04-13

**Authors:** Morin Lang, Stefan Mendt, Valeria Paéz, Hanns-Christian, Gunga, Grzegorz Bilo, Giampiero Merati, Gianfranco Parati, Martina Anna Maggioni

**Affiliations:** ^1^ Department of Rehabilitation Sciences and Human Movement, Faculty of Health Sciences, University of Antofagasta, Antofagasta, Chile; ^2^ Network for Extreme Environment Research (NEXER), University of Antofagasta, Antofagasta, Chile; ^3^ Charité—Universitätsmedizin Berlin, Institute of Physiology, Center for Space Medicine and Extreme Environments Berlin, Berlin, Germany; ^4^ Department of Medicine and Surgery, University of Milano-Bicocca, Milan, Italy; ^5^ Department of Cardiology, Istituto Auxologico Italiano, Istituto di Ricerca e Cura a Carattere Scientifico (IRCCS), Milan, Italy; ^6^ Department of Biotechnology and Life Sciences (DBSV), University of Insubria, Varese, Italy; ^7^ IRCCS Don C. Gnocchi Foundation, Milan, Italy; ^8^ Department of Biomedical Sciences for Health, Università degli Studi di Milano, Milano, Italy

**Keywords:** chronic intermittent hypoxia, high altitude, heart rate variability, hypertension, six-minute walk test

## Abstract

Cardiac autonomic modulation in workers exposed to chronic intermittent hypoxia (CIH) has been poorly studied, especially considering hypertensive ones. Heart rate variability (HRV) has been proven as valuable tool to assess cardiac autonomic modulation under different conditions. The aim of this study is to investigate the cardiac autonomic response related to submaximal exercise (i.e., six-minute walk test, 6MWT) in hypertensive (HT, *n* = 9) and non-hypertensive (NT, *n* = 10) workers exposed for > 2 years to CIH. Participants worked on 7-on 7-off days shift between high altitude (HA: > 4.200 m asl) and sea level (SL: < 500 m asl). Data were recorded with electrocardiography (ECG) at morning upon awakening (10 min supine, baseline), then at rest before and after (5 min sitting, pre and post) the 6MWT, performed respectively on the first day of their work shift at HA, and after the second day of SL sojourn. Heart rate was higher at HA in both groups for each measurement (*p* < 0.01). Parasympathetic indices of HRV were lower in both groups at HA, either in time domain (RMSSD, *p* < 0.01) and in frequency domain (log HF, *p* < 0.01), independently from measurement’s time. HRV indices in non-linear domain supported the decrease of vagal tone at HA and showed a reduced signal’s complexity. ECG derived respiration frequency (EDR) was higher at HA in both groups (*p* < 0.01) with interaction group x altitude (*p* = 0.012), i.e., higher EDR in HT with respect to NT. No significant difference was found in 6MWT distance regarding altitude for both groups, whereas HT covered a shorter 6MWT distance compared to NT (*p* < 0.05), both at HA and SL. Besides, conventional arm-cuff blood pressure and oxygen blood saturation values (recorded before, at the end and after 5-min recovery from 6MWT), reported differences related to HA only. HA is the main factor affecting cardiac autonomic modulation, independently from hypertension. However, presence of hypertension was associated with a reduced physical performance independently from altitude, and with higher respiratory frequency at HA.

## Introduction

In Chile, due to the mountainous geography there is a great variety of working activities at high and very high altitude (HA), i.e., between 2,500 and 5,800 m above sea level (asl) (Parati et al., 2018). Indeed, mines are located for roughly 80% of cases at HA [https://www.sonami.cl/mapaminero/ (Accessed December 2021)]. Thus, because of the need for miners to work in HA, mining companies organize their work by applying a rotating shift system modality, where all individuals’ alternate periods of work at HA and periods of rest at low altitude or sea level (SL) for a time proportional to the time worked at HA. This specific working pattern exposes employees to HA in a unique *chronic* and at the same time *intermittent* manner, which has been defined as chronic intermittent hypoxia (CIH). The most used shift modalities of CIH exposure are 4-on (HA) vs. 3-off (SL) and 7-on vs. 7-off days, the latter being the most frequent, as better compatible with a reasonable quality of workers’ social/private life. According to Chilean Ministry of Health, a worker is considered acclimatized to CIH if he/she has been engaged for more than 6 months in rotating shifts with work at HA (with a minimum of 30% on-duty time) and rest at low altitude or SL (Ministerio de Salud de Chile and Departamento de Salud Ocupacional, 2012).

CIH has been reported as a risk factor for cardiovascular disease (Poulain et al., 2014), however there is still limited information regarding cardiovascular responses to CIH in healthy individuals, as well as in individuals with pre-existing cardiovascular diseases, such as hypertension. In miners, long-term adjustments in relation to ventilatory, cardiovascular, erythropoietic, and physiological responses to CIH tend to be comparable to those observed for chronic hypoxia exposure. In the first days of each shift at HA, an acute response characterized by increase in heart rate (HR) and blood pressure (BP), decrease in blood oxygen saturation (SpO_2_) (Farías et al., 2013), and appearance of acute mountain sickness have been reported (Richalet et al., 2002; Brito et al., 2007). A similar response was recently found also in hypertensive miners (Lang et al., 2021). Some studies indicate that the main cardiovascular effects of long-term exposure to CIH are elevated systemic and pulmonary artery pressure, and right ventricular enlarging (Richalet et al., 2002; Brito et al., 2007; Brito et al., 2018). In addition, Richalet et al. (2002) reported specifically in miners exposed to CIH (sleeping at 3,800 m asl and working at 4,800 m asl, with the 7-on-7-off days shift pattern), a decreased physical capacity and altered sleep patterns after 31 months and a progressive increase in hematocrit (up to 19 months). However, after 31 months of this rotating shift, hematocrit values tend to normalize, returning to pre-exposure levels. In the same study, higher values of BP during the day- and night-time compared to those recorded at SL have been observed, with a tendency for BP measured at HA to decrease over the time (Gunga et al., 1996; Richalet et al., 2002). Recently, a significant increase in conventional and 24 h ambulatory BP has been reported in the first days of shift work at HA; such increase tended to be more accentuated in hypertensive compared to normotensive individuals (especially during the night), despite treatment (Lang et al., 2021). In such a context, in 2017 clinical hypertension was reported to affect 13.8% of the mining population with 7-on and 7-off days shifts in Chile, resulting in an important increase in cardiovascular risk for this population (Escuela de Salud Pública and Universidad de Chile, 2018). Furthermore, the higher hypoxia-driven response of BP to submaximal and maximal exercise in hypertensives subjects exposed to acute HA might be accompanied by a lower threshold for coronary artery disease symptoms (Caravita et al., 2014; Caravita et al., 2015; Lang et al., 2016). Changes in the physiological responses to maximal and submaximal exercise at HA (chronic exposure) have also been documented, especially in adult individuals (Fulco et al., 1998; Mazzeo, 2008). Submaximal exercise performance is known to be reduced at HA, due to a lower inspired oxygen pressure, whose main effect is the drop in arterial oxygen content and its availability in the tissues, displayed by decreased arterial oxygen saturation in the first 24 h of HA exposure (Buskirk et al., 1967; Beall, 2007; Grocott et al., 2009). Therefore, long term CIH might also be associated with a decreased functional capacity in miners. The six-minute walk test (6MWT), a simple and inexpensive test, is largely employed in several clinical settings to evaluate functional status and hemodynamic responses to submaximal exercise (Solway et al., 2001; Crapo et al., 2002). The few studies that investigated submaximal exercise and hemodynamic acute response to HA conducting the 6MWT, indicated that it has a good safety profile in healthy individuals (Lazio et al., 2010) as well as in cardiac (Vona et al., 2006), and in hypertensive patients, with and without pharmacological treatment (Lang et al., 2016). This was the case also after HA chronic exposure (Caffrey et al., 2014). In this context, cardiac autonomic modulation assessment might be helpful to get a better insight into the mechanisms underlying the changes in physical performance reported at HA. This could be the case for the population of miners exposed to CIH with the 7 × 7 days pattern over long time periods. Heart rate variability (HRV) is a non-invasive tool known since long time to be able to assess cardiac autonomic modulation. HRV represents an informative tool for all pathological conditions where autonomic derangements typically develop, such as arterial hypertension, diabetes (Parati et al., 1995; Villafaina et al., 2017; Maciorowska et al., 2020; Yılmaz et al., 2020) and heart failure (Hsu et al., 2015; Tsai et al., 2020). Moreover, HRV has been proposed as a monitoring/predicting tool for estimating the level of athletic performance (Plews et al., 2013). Recently, a predictive role of HRV in pre-hypertensive patients has been suggested (de Oliveira et al., 2020). Furthermore, a huge body of literature has been published proving the role of HRV as an independent predictor for cardiovascular disease and sudden cardiac death (Thayer et al., 2010; Huikuri and Stein, 2013; Sessa et al., 2018). Indeed, HRV assessment has been implemented in several fields of research and clinical areas, and has been shown to provide insights into effects of exercise on the heart in several conditions, from health to disease (Besnier et al., 2017; Raffin et al., 2019; Figueiredo et al., 2020). Therefore, also in the conditions of our study the assessment of HRV might be very useful investigate the impact of long term CIH exposure on cardiac autonomic modulation of miners, especially in those with pre-existing cardiovascular conditions such as hypertension. So far, to the best of authors’ knowledge there are no published studies dealing with this topic. The only studies available have been conducted in animals’ models (Rey et al., 2008; Lucking et al., 2018; Shi et al., 2020), and only a few in humans (Almeida et al., 2017; Morand et al., 2018), but with a completely different pattern of CIH and employing simulated altitude techniques (Iturriaga et al., 2009; Chacaroun et al., 2017; Lizamore and Hamlin, 2017).

As the exposure to high altitude and intense physical exercise have both been commonly considered to potentially increase the cardiovascular risk in patients with arterial hypertension, we decided to explore the possible interaction of pharmacologically treated hypertension with hemodynamic adaptation (along with its autonomic modulation) to CIH exposure in a professional group of hypertensive miners. Indeed, arterial hypertension is well represented in these workers, and it is therefore necessary to assess whether intermittent exposure to high altitude combined with submaximal physical exercise might carry a higher occupational risk profile in this professional category (Aragón-Vela et al., 2020). The aim of this study is therefore to explore the cardiac autonomic response in the morning (upon awakening), before and after submaximal exercise (i.e., six-minute walk test, 6MWT) in treated hypertensive (HT) and in non-hypertensive (NT) workers exposed for longer than 2 years to CIH with the rotating shifts pattern 7 × 7 days. We hypothesized that i) during the first day of exposure at HA cardiac autonomic modulation still depict a strongly reduced vagal outflow with respect to SL, possibly independently from hypertension, ii) HT individuals show lower physical performance with respect to NT ones, especially at HA, considering HRV and HR recovery, and iii) mechanisms and timing of acclimatization might differ between HT and NT.

## Methods

### Subjects

**FIGURE 1:**
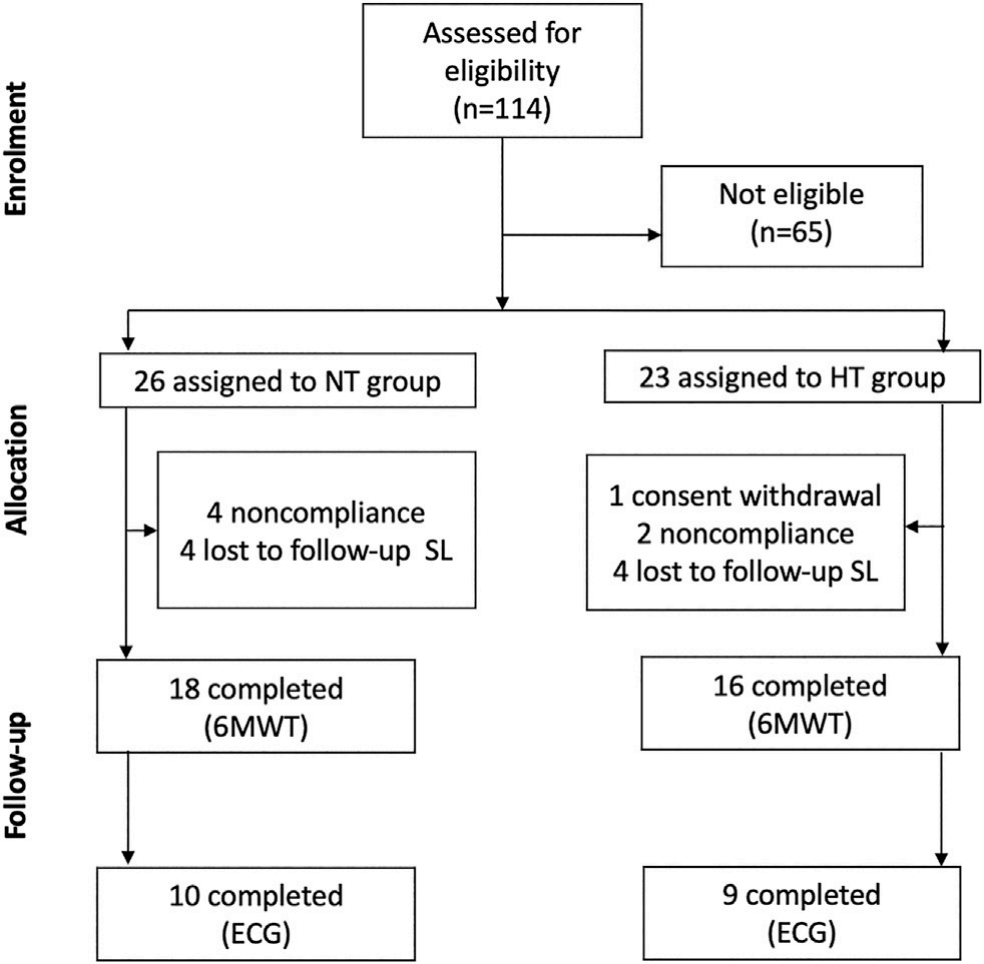
Study design. Flow chart diagram representing the enrollment and the experimental procedure. NT: non-hypertensive; HT: hypertensive; SL: sea level; 6MWT: six minutes walking test; ECG: electrocardiography.

A total of 34 men were initially enrolled in this observational, cross-sectional study. The participants are a subsample of a larger study from the same research team and were selected according to the procedure reported by Lang et al. (2021). All individuals were employees of a mining company in northern Chile for at least 2 years. Briefly, criteria for eligibility were: 19–60 years of age, body mass index (BMI) <35 kg/m^2^, a permanent residence at low (< 500 m asl) altitude, no hypertension history (i.e., no pharmacological treatment for hypertension, NT group) or presence of hypertension, but on treatment (HT group). Participants with cardiovascular diseases other than hypertension, or with suspected or confirmed secondary hypertension, diabetes mellitus, as well as those with hypertension on treatment with beta-blockers, were excluded from this specific study. Subjects’ information details were initially obtained from an internal database of a health occupational quality of life program of the mining company, made available to the researchers. Inclusion and exclusion criteria were then applied, selecting a total of 49 workers, of which 26 were assigned to NT and 23 to HT groups; out of them (*n* = 49), 18 NT and 16 HT completed the 6MWT (*n* = 34), and 10 and 9 respectively completed the ECG recording as well (Figure 1). The physical workload of the miners included in this study is generally low and moderate in both groups. The jobs were for both groups mainly mine operator (i.e., drivers, 60%), then administrative employee (e.g., supervisors, draftsmen, engineers, etc., about 30%) and maintenance worker (mechanics and electricians, 10%). After detailed explanations by the investigators, all participants gave a written informed consent to their participation. The study was approved by the Ethics Committee of the University of Antofagasta (ethic approval number: 181/2019), and conducted in agreement with the Declaration of Helsinki (World Medical Association, 2013).

### Experimental Procedure

**FIGURE 2:**
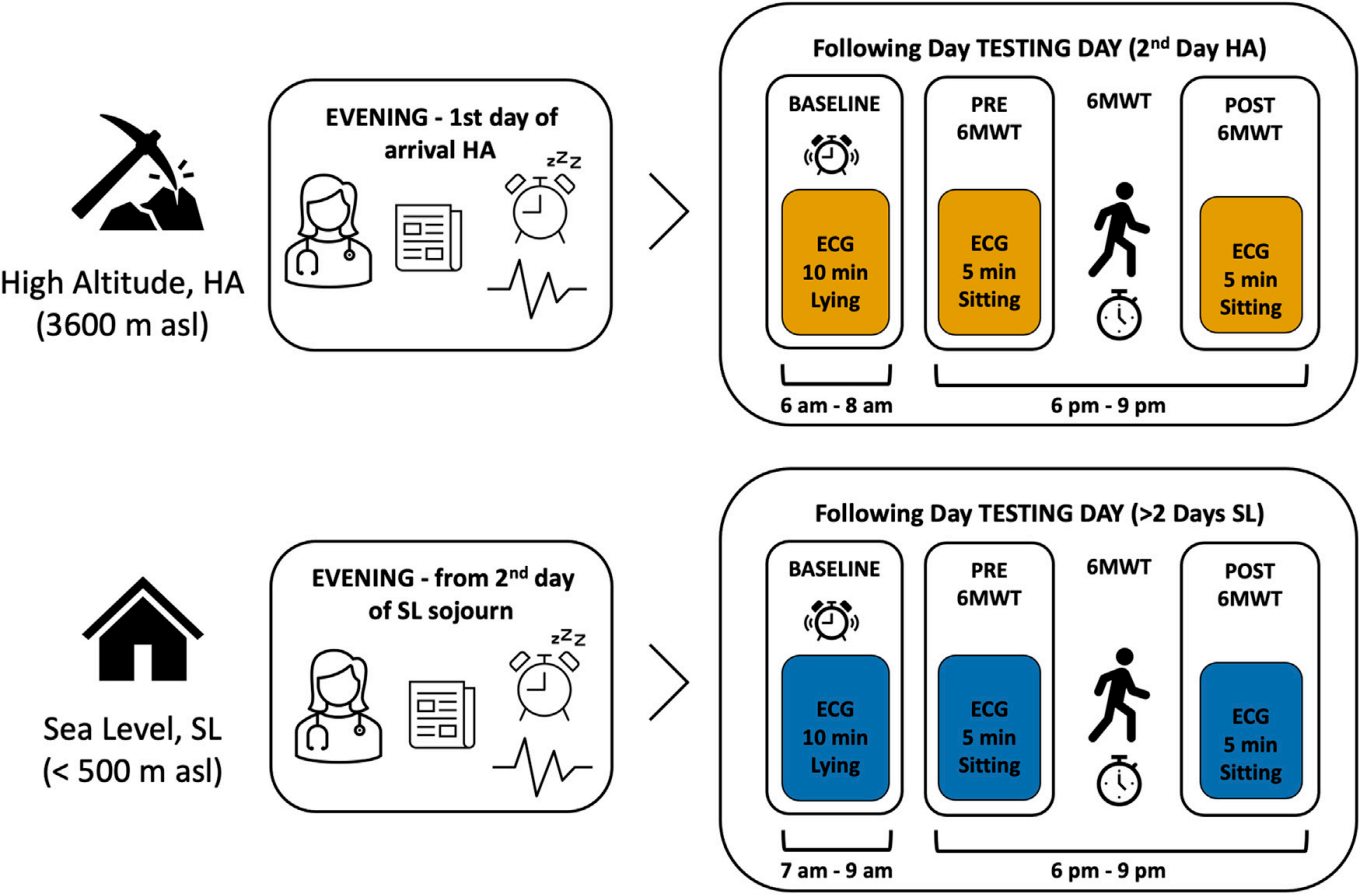
Experimental protocol overview. Schematic description of experimental protocol. Measures at SL and HA were performed in a random sequence. On the evening of their arrival in HA (boxes to the right) participants were instructed by the researcher (pictogram doctor) on the use of the wearable device for measuring the electrocardiography (i.e., ECG, pictogram line as ECG wave) and were informed on the recording constraints. They also signed the informed consent (pictogram document) and were asked to note the time of awakening in the following morning (pictogram alarm clock). Once instrumented with the ECG sensor, the continuous monitoring was started and they were asked to go to sleep. During the following day (testing day), from the continuous ECG recording, specific time windows were selected for data analysis: in the morning, upon awakening, a time window of 10 min (Baseline) and two time windows of 5 min each, before (PRE) and after (POST) the 6-minute walking test (6MWT), see boxes to the left.

As above mentioned, the detailed description of screening and enrolment procedure is reported by Lang et al. (2021). Participants selected to be eligible, entered the study after medical history was taken and following pharmacological treatment assessment. Measurements took place at two different time points according to equipment availability and will/availability of miners, 1) during the first 2 days upon arrival at HA for the 7-on days’ work shift, and 2) after the second day of sojourn at low altitude (< 500 m asl), during the 7-off days period, which is considered hereafter as sea level (SL). For an overview of experimental protocol see Figure 2.

During the work shift miners sleep at 3,800 m asl, while work activity is performed between 4,203 and 4,797 m asl, in 7-on−7-off days shifts. Upon arrival at the hotel, in the evening, participants were instructed about measurements procedures: the investigator showed all details regarding maintenance and function of the miniaturized, wearable, 1-lead electrocardiography (ECG) device, to be worn continuously for the following 36 h, allowing short interruption for personal hygiene needs. Participants were informed about the functioning of the ECG device: they were asked to set a marker by shortly pressing the on-off button in the morning upon awakening, while resting in bed in the supine position. They were requested to avoid caffeine and alcohol consumption, and extra physical training during the whole recording. On the following day, once returned to the hotel after work, participants underwent a 6-min walk test (6MWT). The 6MWT was performed for all participants in the identical setting, same track, delimited by cones, same ambient temperature and illumination, according to the reference procedure (Crapo et al., 2002). After allowing a sitting resting phase of at least 5 min, participants were instructed about the test procedure and the same operator conducted the test under strict monitoring and annotations of results. After 6MWT, passive recovery sitting was scheduled for 5 min. The pre-test and post-test phases were recorded in the ECG by setting a marker. Furthermore, on the same day, details about bed and waking time, working schedule and eating time were collected by personal interview from the operator.

As for the data collection at SL, the same operator visited participants at their homes, planning the appointment within the second and the fifth day of sojourn. ECG data collection was conducted according to the same procedure implemented at HA, including personal interview. 6MWT was performed at the same time of day, with identical procedure, by selecting a track delimited by cones, with similar conditions as at HA.

### Six-Minute Walk Test

The 6MWT was performed according to the American Thoracic Society protocol (Crapo et al., 2002). Before starting the test, participants were resting seated for at least 5 min, then, prior to test performance, HR and BP (Systolic Blood Pressure-SBP and Diastolic Blood Pressure-DBP) were recorded with a validated oscillometer device (AND UA-767Plus, Japan), together with peripheral oxyhemoglobin saturation (SpO_2_), measured on the finger with a pulse oximeter (Vantage 9590, NONIN, USA). The same measurements were performed immediately at the end of the 6MWT and after 5 min of passive recovery in seated position. The test was conducted in a corridor 30-m long with the floor marked every 3 m. by means of an adhesive tape. Two trained physicians supervised and conducted the test at sea level and at high altitude. All the individuals had previously familiarized with the test and were aware of the procedure. During the test, standard encouragement to maintain a high-performance level was given in the Spanish language every minute. Operators recorded the 6MWT results, in terms of distance covered in meters and cardiorespiratory variables response.

### Heart Rate, Heart Rate Variability and Heart Rate Recovery

ECG traces were recorded with a miniaturized wearable 1-lead electrocardiography (ECG, Bittium Faros 180, Finland). The device is very small, light, and smoothly designed to ensure individual comfort, even during sleep, and it doesn’t interfere with regular daily activities, including work and exercise.

Both recently and in the past, several papers have been published about the standards and the physiological meaning of HRV indices (Parati et al., 1995; Task Force of the European Society of Cardiology and the North American Society of Pacing Electrophysiology, 1996; Sassi et al., 2015; Shaffer and Ginsberg, 2017; Singh et al., 2018).

For this study, main outcomes are 6MWT distance and heart rate (HR), as well as HRV indices. Specifically, due to short-term HRV analysis applied to the selected time windows, we considered: 1) in the time domain, the RMSSD (i.e., the Root Mean Square of Successive Differences of NN- normal-to-normal beats intervals in ms) as index of vagal activity 2) in the frequency domain the Total Power of spectral analysis (TP), describing the amount of variability, and the log HF (i.e., High Frequency power, 0.15–0.4 Hz), which expresses the vagal influences on HR and includes respiratory sinus arrhythmia. Moreover, two indices in non-linear domain were considered: short-term fractal index by detrended fluctuations analysis (detrended fluctuation alpha 1, DFA1), which represents a vagal-sympathetic balance index (Tulppo et al., 2001; Castiglioni et al., 2011), not affected by respiration, and the sample entropy (SampEn), an index which describes the complexity and the regularity of the signal.

To gain a better understanding of the above mentioned outcomes, mean respiratory frequency has to be considered, thus ECG-derived respiration (Sakai et al., 2019) was also estimated. ECG-derived respiration reflects the movements of the thoracic grid during respiration and it is assessed as modulated amplitude of QRS complex (Schmidt et al., 2017; Sakai et al., 2019; Varon et al., 2020).

Furthermore, we focused on heart rate recovery (HRR) after the 6MWT, as a marker of cardiovascular fitness and its dynamic adaptation, analyzing HR values after 30 s (HRR_30_) and after 60 s (HRR_60_) from the 6MWT end (i.e., HRmax).

### Data Analysis

An expert operator visually inspected the ECG signal, identifying and manually removing possible artefacts and premature beats. A cut-off artefacts rate of 3% of total beat number was set to consider data quality of each series satisfactory enough to enter further processing for HRV. Beat-by-beat time series of normal-to-normal R-R intervals (i.e., N-N series) were then derived from the original ECG tracing (sample rate 250 Hz) for HRV analysis. In the time domain we considered HR and RMSSD (Root Mean Square of Successive RR interval Differences), index of vagal tone. The power spectrum of NN intervals was calculated (FFT spectrum, window width of 300 s and a 50% overlap between windows) to derive the total spectral power (TP) and the spectral power in the high frequency range (HF, from 0.15 to 0.40 Hz). Furthermore, the non-linear domain indices DFA1 and SampEn were computed (see above). The mean breathing rate was evaluated with the ECG-derived respiration (EDR) method. All the analysis were performed with the software *Kubios* ver. 3.4.2 (Kuopio, Finland) (Tarvainen et al., 2014). Heart rate recovery (HRR) was assessed on the 5-min passive recovery phase (sitting) immediately following the end of 6MWT by calculating HRR_30_ and HRR_60_ metrics (defined by the difference between HRmax at the end of the 6MWT and HR after, respectively, 30 and 60 s recovery), according to Peçanha et al. (2017).

### Statistics

Separated linear mixed-effect models were run with *Subject* as a random effect (random intercepts) and *Altitude* (HA, SL), *Group* (NT, HT), *Time,* and their interactions as fixed effects. Covariance matrices were determined by restricted maximum likelihood estimation. For punctual measures (HR, SpO_2_, SBP, and DBP), factor *Time* included levels “Start” (begin of 6MWT, min “0”), “End” (immediately after the end of 6MWT, min “6^th^”), and “5 min recovery” (5 min after 6MWT, min “11^th^”). For HRV indices, *Time* levels were “base” (morning upon awakening, 10-min supine), “pre” (before 6MWT, 5-min sitting), and “post” (after 6MWT, 5-min sitting). Changes in 6MWT Distance, HRR_30_, and HRR_60_ were analyzed by linear mixed-effect models with *Altitude* (HA, SL), *Group* (NT, HT), and their interactions as fixed effects, and *Subject* as a random effect. When effect for *Group*, *Altitude*, or interactions was significant, contrasts were used to quantify these effects. If necessary, contrasts were adjusted for multiple comparisons using family-wise error rate procedure by Hochberg (Hochberg, 1988). We checked normality assumption by analyzing the model residuals (visual inspection of Q–Q plots). The level of significance was set at *α* = 0.05 (two-sided) for all testing. Linear mixed-effect models were run using the *afex* package (Singmann et al., 2020). Estimated marginal means with standard errors (EMM ± SE) and contrasts were calculated using the *emmeans* package (Lenth, 2020). All statistical analyses and graphical illustrations were performed using R (R Core Team, 2019).

## Results

All 34 enrolled participants underwent the 6MWT at both HA and SL. However only 30 ECG traces, ca. 36-h long, were collected, as 4 participants failed to complete recordings. A total number of 90 datasets were selected, corresponding to three time points (i.e., baseline, pre and post 6MWT) per each participant. After careful quality evaluation of collected data, by an expert operator, 57 datasets were found eligible for further analysis to assess HRV, with full data and sufficient quality for all the three time points.

The reasons for excluding a total number of 33 datasets (corresponding to 11 participants) were: low-quality or incomplete ECG traces for the three selected time windows (i.e., artefacts or ectopic beats exceeding the 3% of the whole recording, or incomplete recordings).

The final sample entering HRV analysis and considered in this study comprises a total number of 19 participants (NT, *n* = 10; HT, *n* = 9). Table 1 reports sample’s descriptive data. NT and HT groups did not differ significantly in terms of age, BMI and working experience, as well as altitude of workplace. Regarding anti-hypertensive therapy of HT participants: *n* = 4 were treated with Angiotensin II receptor blockers (ARBs); *n* = 2 with combinations of angiotensin II receptor blockers and calcium antagonist; *n* = 2 with calcium antagonist (CA); *n* = 1 with Angiotensin-Converting Enzyme inhibitor (ACE inhibitors). No treatment changes occurred between SL and HA condition.

**TABLE 1:** Sample’s demographic characteristic.

	HT	NT	*p* values
**Anthropometry**			
Age (years)	50.7 ± 8.0	44.8 ± 6.3	0.110
Body mass index (kg⋅m^−2^)	27.2 ± 3.4	29.6 ± 2.1	0.141
**Working experience**			
Workplace location (altitude, m)	4533 ± 313	4467 ± 362	0.712
Career duration (years)	11.18 ± 4.84	10.53 ± 4.52	0.792

Descriptive data are reported as mean ± standard deviation. Differences between the HT (n = 9) and the NT (n = 10) group were assessed by Wilcoxon’s signed rank test.

**FIGURE 3:**
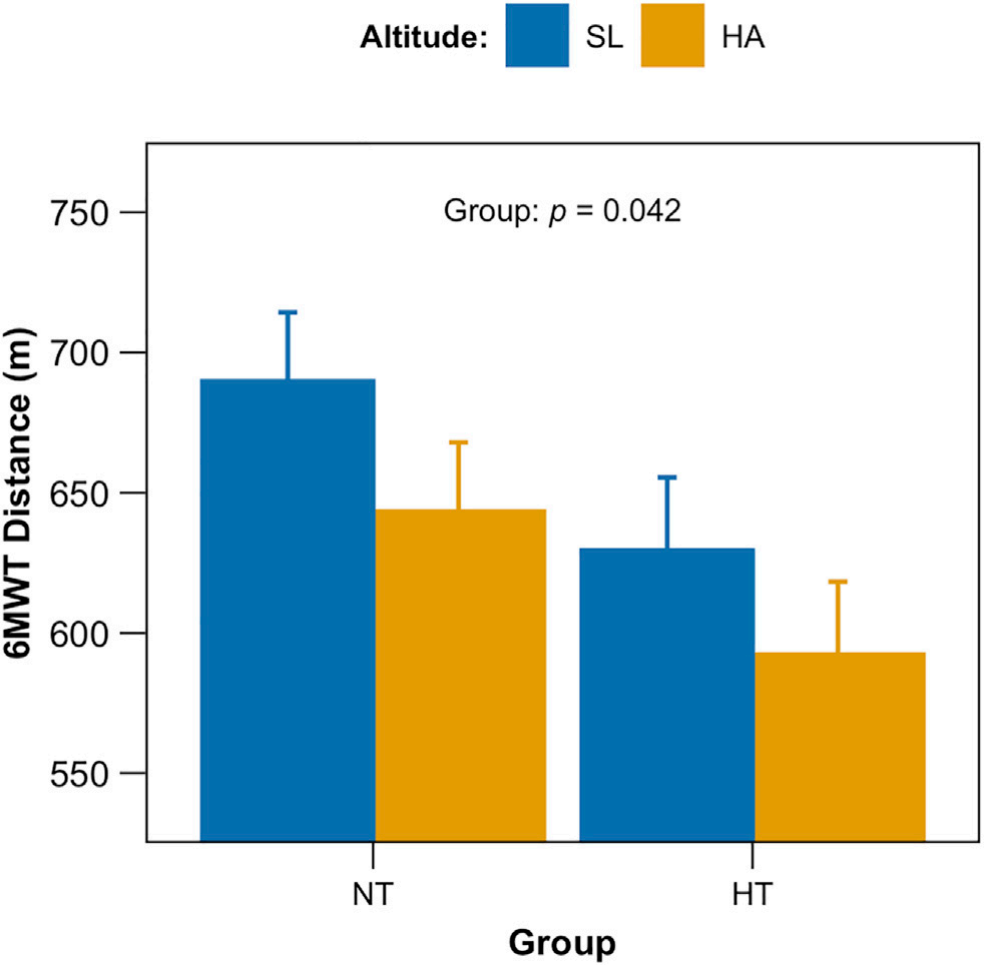
Six-minute walk tests (6MWT) results. Total distance covered (in meters) after the 6MWT for both groups (NT, non-hypertensive; HT, hypertensive) at both altitudes (*p* = 0.042 for group).

Overall, the data relating to distance covered in the 6MWT at SL do not differ much from a population norm based on weight, height, sex and age, predicted according to the Enright formula (Enright and Sherrill, 1998). In fact, the deviation from the norm was found to be equal to 5 ± 15% (m ± SD). As reported in Figure 3, 6MWT results did not differ significantly by altitude (*p* = 0.117), whereas a significant difference was found between groups: independently from altitude NT performed better than HT: HA, 643 ± 24 vs. 592 ± 26 m; SL 690 ± 24 vs. 629 ± 26 m (EMM ± SE), NT vs. HT respectively (*p* = 0.042).

**FIGURE 4:**
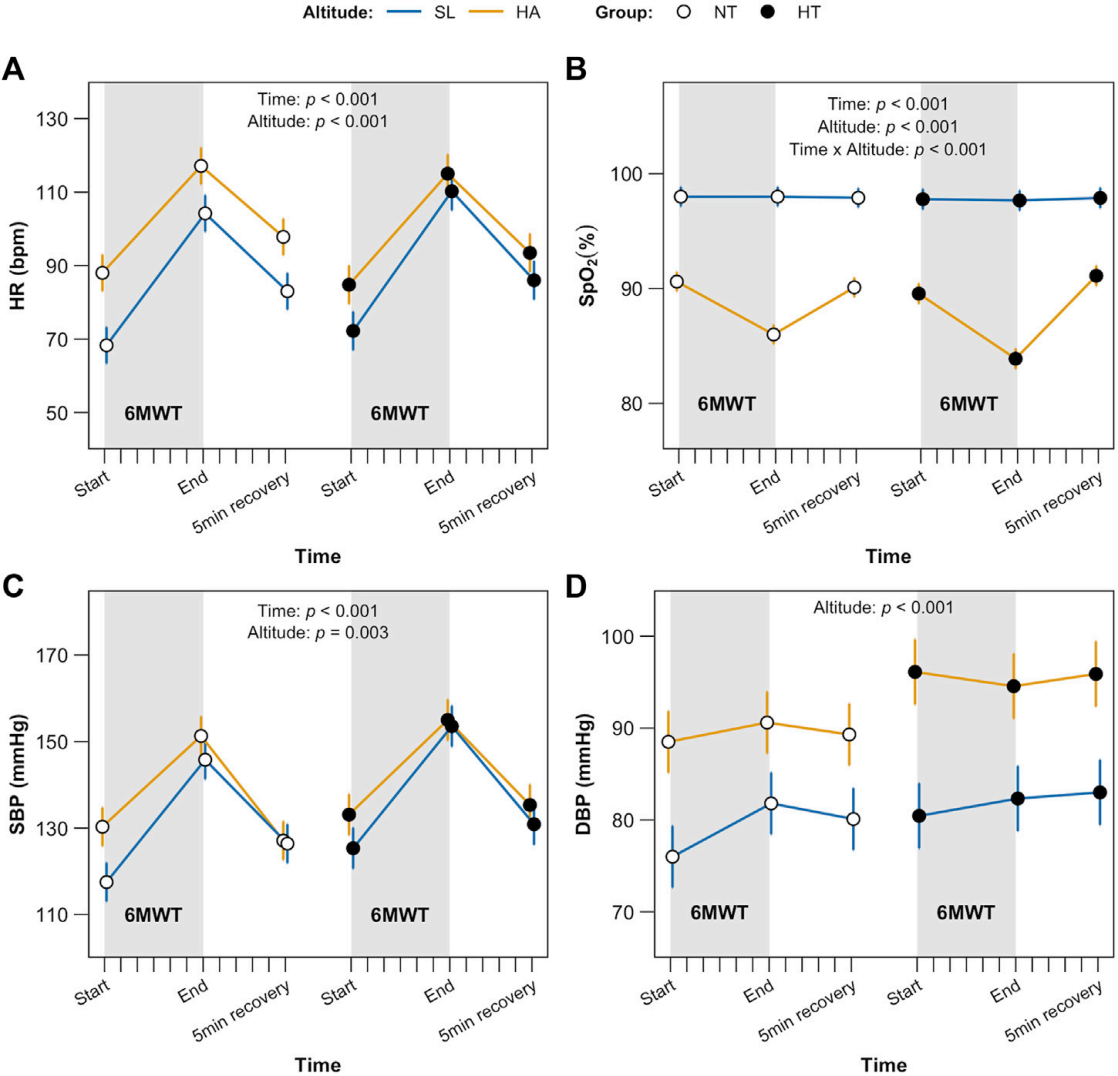
6MWT complimentary data. Punctual measure of heart rate, HR **(A)**, blood oxygen saturation, SpO_2_, **(B)**, systolic blood pressure, SBP **(C)** and diastolic blood pressure, DBP **(D)**, immediately before starting the 6MWT (= min 0, Time), at the end (= min 6^th^, Time) of 6MWT and after 5-min recovery (= min 11^th^, Time). Significant factors and interactions were HR and SpO_2_ time and altitude (both *p* < 0.01); SpO_2_ time and altitude (*p* < 0.001). SBP time and altitude (*p* < 0.001 and *p* = 0.003 respectively); DBP altitude only (*p* < 0.001).

Furthermore, punctual measures of HR, SpO_2_ and SBP/DBP collected prior 6MWT, immediately at its end and after 5-min recovery sitting, are displayed in Figure 4. Considering HR (Figure 4A) a main effect for both time and altitude (*p* < 0.001) was retrieved as well as for SpO_2_ (Figure 4B), with also the interaction time x altitude (*p* < 0.001 for all). SBP (Figure 4C) showed an effect of time and altitude (*p* < 0.001and *p* = 0.003 respectively), whereas DBP (Figure 4D) showed an effect of altitude only (*p* < 0.001). No significant differences were found as for the factor group.

HRpeak values (at the end of the 6MWT) for HRR_30_ and HRR_60_ calculation were for SL: NT 130 ± 5 vs. HT 129 ± 5 bpm; for HA: NT 148 ± 5 vs. HT 141 ± 5 bpm (EMM ± SE, *p* = 0.003 for altitude only). HRR_30_ analysis reported a significant main effect for group (*p* = 0.027) and for altitude (*p* = 0.036); follow up comparison looking at the interaction between altitude and group revealed that this difference was related to SL [NT 25.9 ± 2.8 vs. HT 15.6 ± 2.9 bpm (EMM ± SE), *p* = 0.035]. As for HRR_60_, no significant main effect was retrieved.

**FIGURE 5:**
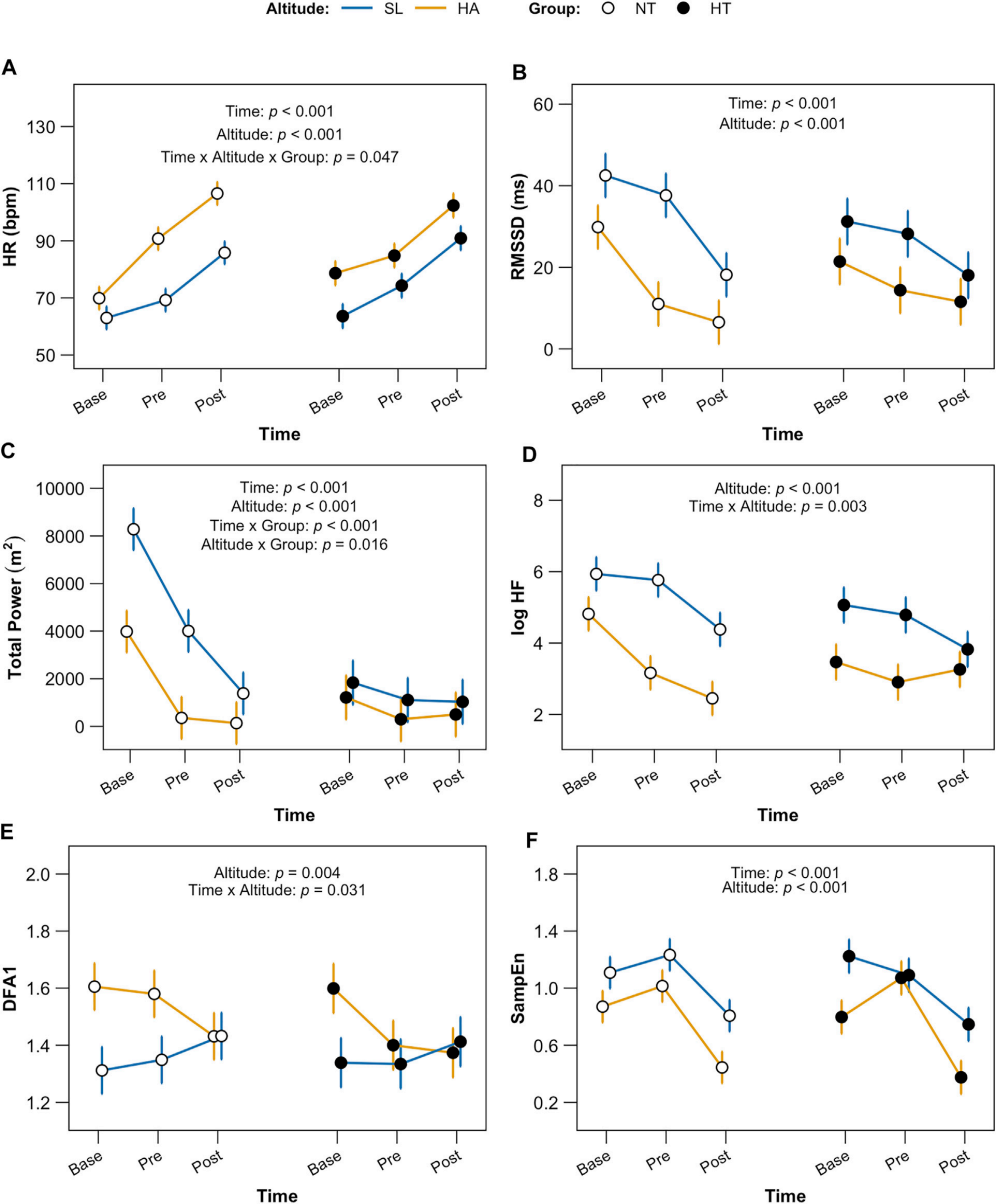
HRV indices. Data are displayed per each time point (baseline, morning upon awakening, supine; pre, before 6MWT sitting; post, after 6MWT, sitting) for both groups (NT and HT) at both altitude (HA and SL). Panel **(A)** shows HR (heart rate); statistical significance: factors time and altitude *p* < 0.001, time x altitude x group *p* = 0.047. Panel **(B)** shows RMSSD (root mean square of successive differences between normal heartbeats), an index of vagal tone in time domain; factors time and altitude: *p* < 0.001. Panel **(C)** shows TP (total power spectrum density); factors time and altitude: *p* < 0.001, group *p* = 0.007; time x groups *p* < 0.001 and altitude x group *p* = 0.016. Panel **(D)** shows logHF (High Frequency power), index of vagal outflow and RSA in frequency domain, altitude *p* < 0.001, time x altitude *p* = 0.003. Panel **(E)** displays DFA1 (detrended fluctuation alpha 1), index of vagal-sympathetic balance in non-linear domain, altitude *p* = 0.004 and time x altitude *p* = 0.031 and Panel **(F)** shows SampEn (Sample Entropy), factors time and altitude *p* < 0.001.

Mean HR recorded by ECG at baseline (morning upon awakening, 10 min, supine), before and after 6MWT (5 min, sitting) at HA was significantly higher in comparison with SL, per each time points in both groups (NT and HT, *p* < 0.001 for both time and altitude). The interaction time x altitude x group was found significant (*p* = 0.047) (Figure 5A). HRV index in the time domain, RMSSD, showed a significant reduction in both groups at HA (*p* < 0.001 for both time and altitude), also mirroring the expected difference between baseline and pre/post 6MWT (higher vagal tone at morning upon awakening), independently from group allocation (Figure 5B).

HRV indices of variability and vagal tone in the frequency domain, TP and log HF, showed a similar pattern with significant decrease (*p* < 0.001 for both time and altitude). However, as with TP, a strongly significant interaction time x group (*p* < 0.001) was identified: TP was significantly lower at any time point also at SL level for HT in comparison with NT (Figures 5C,D).

Results in non-linear domain displayed for DFA1 a significant effect of altitude (*p* = 0.004) and time x altitude (*p* = 0.031), with higher values at HA and at morning (baseline); the SampEn showed a significant reduction in complexity at HA and especially in the morning (baseline), *p* < 0.001 for both time and altitude (Figures 5E,F).

**FIGURE 6:**
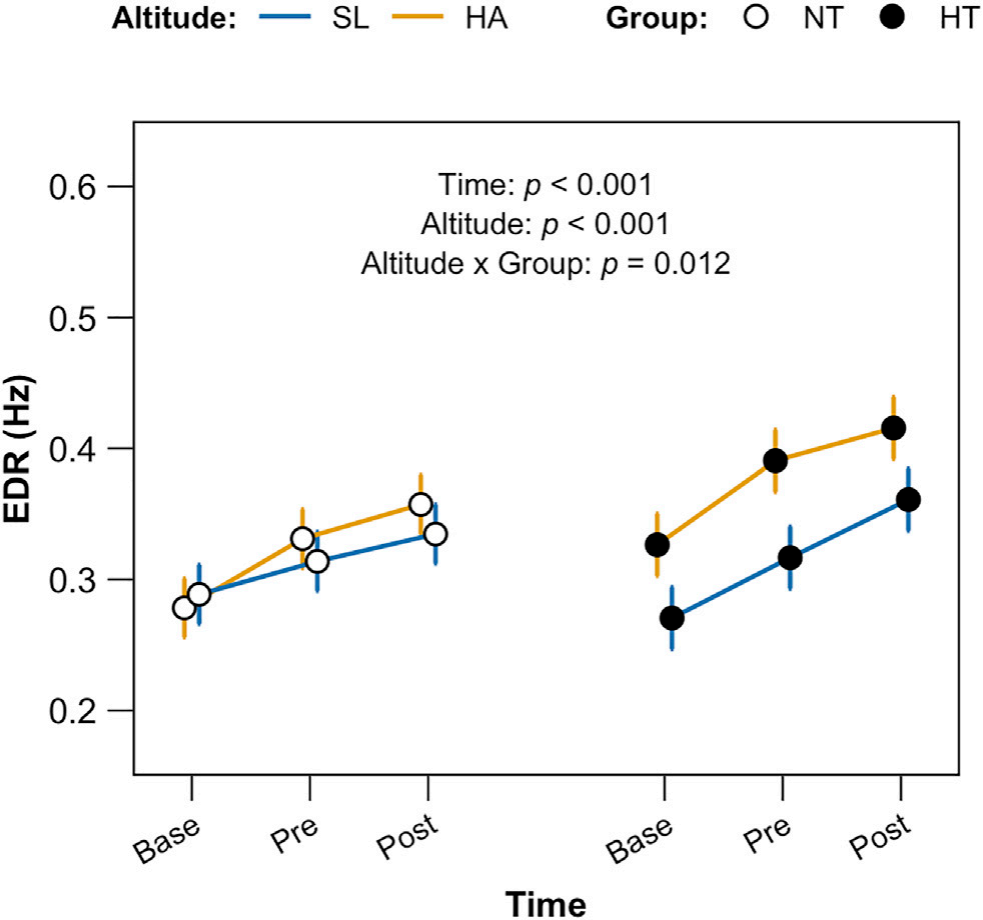
ECG—derived respiration (EDR) data. Data are displayed per each time point (baseline, morning upon awakening, supine; pre, before 6MWT sitting; post, after 6MWT, sitting) for both groups (NT and HT) at both altitudes (HA and SL). Factors time and altitude *p* < 0.01; altitude x group *p* = 0.012.

EDR data (Figure 6) depicted a significant effect for both time and altitude, with higher HA respiration rate per each time point in both groups (*p* < 0.001). A significant interaction for time x group was found (*p* = 0.012).

## Discussion

Our study provides evidence of important changes in cardiac autonomic modulation in individuals exposed to CIH, either in presence of normotension and treated hypertension. First, workers exposed to CIH through 7-on-7-off days’ work shift rotation, even after several years seemed not to be completely acclimatized. Data collected upon awakening in the first morning after arrival at HA, still showed depressed HRV and vagal tone, reduced signal complexity, as well as higher respiratory frequency, independently from the blood pressure condition. Secondly, a submaximal physical test, the 6MWT, showed reduced performance of HT both at HA and SL. Third, HT individuals showed a larger effect of HA on HRV, especially in the morning upon awakening (baseline), as well as on respiratory rate and fast part of HR recovery (i.e., HRR_30_) after submaximal exercise. To the best of author’s knowledge this is the first work dealing with the topic of cardiac autonomic modulation effects of CIH both at rest and in conditions of sub-maximal physical performance. So far, previous studies published on cardiac autonomic modulation and CIH dealt with a completely different CIH pattern (Fu et al., 2007; Chacaroun et al., 2017; Lizamore and Hamlin, 2017) consisting in few hours of exposure-non exposure, and based on use of simulated hypobaric/non-hypobaric hypoxia, or on animals’ models (Rey et al., 2008; Lucking et al., 2018; Shi et al., 2020), again with different CIH patterns. On the contrary we focused here on a 7 × 7 days CIH pattern, with HA exposure of humans for a very long period (>2 years). Furthermore, we considered a very crucial factor for occupational medicine, by focusing on workers with pre-existing cardiovascular conditions, such as hypertension, frequently observed in this specific age category (40–60 years) (Wang et al., 2020).

Considering our results related to data collected for cardiorespiratory parameters (HR, SpO_2_ and SBP/DBP at 6MWT start, at its end and after 5-min recovery), the expected effect of HA was evident in both normotensive and treated hypertensive individuals as acute response to HA: SpO_2_ was significantly decreased (Richalet et al., 2002; Brito et al., 2007; Moon et al., 2016) at each time point, with respect to SL, whereas HR was higher. SBP/DBP results are also in line with previous studies in hypertensive individuals (Bilo et al., 2015; Caravita et al., 2015; Lang et al., 2016), showing that the anti-hypertensive treatment was effective in reducing the enhanced blood pressure response to exercise in hypertensive individuals exposed to HA. Therefore, the anti-hypertensive treatment seems to be effective in counteracting cardiovascular increased response at HA as well. At a first glance, HA appears the crucial factor responsible for reduced functional capacity, by exerting a similar effect on both groups, independently from the presence of hypertension. This observation also allows us to emphasize, once again, the important protective role of an effective antihypertensive treatment in hypertensive workers who must carry out their professional activity during acute exposure to HA.

On the other hand, as expected 6MWT results showed significant lower distance covered by HT individuals with respect to NT, not only at HA but at SL as well (group *p* = 0.042). According to the literature, and 6MWT distances of HT individuals around the 50th percentile of Chilean healthy men age-matched (Osses et al., 2010; Casanova et al., 2011), both at HA and at SL. Therefore, treated hypertension seems to be associated with a reduction in functional capacity, independently from altitude. The role of ARB and ACE inhibitor treatments may be ruled out in our study conditions, as it has been shown that these pharmacological interventions can even improve performance in 6MWT in treated versus untreated individuals (Coelho et al., 2016). Additional factors, may have played a role regarding functional capacity in this specific population, as for example, a more pronounced sedentary lifestyle of hypertensive individuals in comparison with non-hypertensive ones has been reported (Esenamanova et al., 2014).

The HRV analysis provided additional insights into the mechanisms behind the reduced functional capacity observed in HT individuals during the sub-maximal exercise tasks. First, HR in the morning (baseline) and at each time point was higher at HA than at SL in both normotensive and hypertensive individuals. The significant interaction we found among time, altitude and group depicts the different adaptation to HA of HT with respect to NT. In HT, HR was higher in the morning and its increase in pre-6MWT phase with respect to baseline values was lower than in NT. In HT, HR was higher in the morning and its increase in pre-6MWT phase with respect to baseline values was lower than in NT. Regarding the parasympathetic indices of HRV (RMSSD and log HF), we observed a parallel decrease in both the NT and HT individuals (with no significant differences between groups) of these indices when going from rest, pre-exercise, and recovery conditions. This suggests that acute exercise adaptation mechanisms (sympathetic activation and parasympathetic deactivation) are preserved in both NT and HT individuals, even during HA exposition. We are not able to clarify whether the lack of difference between groups might be due to the effects of antihypertensive treatment, because we have not evaluated the behavior of the hemodynamic parameters at HA in the absence of the treatment. However, the similar hemodynamic adaptation of HT and NT individuals to 6MWT allows us to hypothesize that there is no major deviation in the autonomic adaptation mechanisms to exercise even at HA in hypertensive patients.

Considering post-exercise recovery, the typical indices of HRR_30_ (rapid recovery due to vagal reactivation) reported a significant difference between groups at SL only, where the difference between the HRmax and the HR after 30 s of recovery was almost double in NT. As for HT, values were similar in both conditions, HA and SL. HA exposure appears to have masked the difference between NT and HT individuals in parasympathetic fast adaptation (i.e., reactivation) at the end of exercise, given that at HA in NT individuals this adaptation was slowed down. As for HRR_60_ (mixed recovery due to both sympathetic deactivation and parasympathetic reactivation), this index did not show significant differences between groups, still suggesting all in all a similar and equally efficient hemodynamic mechanisms, probably as a consequence of the effective antihypertensive treatment.

Differently, the complex HRV indices DFA1 and SampEn did not changes between groups in all conditions, but showed a non-linear adaptation to altitude and exercise, suggesting a disequilibrium occurring in the sympathovagal balance. DFA1, which represents the balance between the two brunches of autonomic nervous system (i.e., parasympathetic and sympathetic; showing an increase when the sympathovagal balance increases or the vagal tone decreases), was higher at HA in both groups with respect to SL, and the interaction between altitude and time showed that it was higher especially in the morning upon awakening and at pre-6MWT during the first day, whereas after the 6MWT it was similar to SL values. However, how much such imbalance might account for the tachycardia at rest and for the limited adaptation of HR to exercise at HA remains difficult to understand and needs further consideration.

We collected data on HRV in the morning upon awakening at both HA and SL. These data support the usefulness of analyzing baseline cardiac autonomic modulation to describe the acute effect of HA (following the first night at HA) in both groups. First, HR in the morning (base, Figure 5A) was higher at HA than at SL in both groups. It is interesting to note that, again in resting conditions, the acute adaptation to high altitude (HA), which is known to produce a strong cardiac stress also in normotensive individuals, induced similar variations in the hemodynamic parameters in both groups of miners. The hemodynamic variations of acute altitude adaptation are well described in literature and our data go in the expected direction: HR acutely increased in both HT and NT groups, in association with the increase in cardiac work, to match the reduced arterial O_2_ content. Indeed, it has been demonstrated that the rise in cardiac output as a response to acute HA exposure is almost entirely explained by an increase in HR (Naeije, 2010). As such reaction is usually more evident in hypertensive individuals (Caravita et al., 2015), it is therefore conceivable that in our hypertensive group of miners the pharmacologic treatment reduced the abnormal hemodynamic reactions typical of this condition upon exposure to high altitude. This is clinically relevant for the occupational cardiovascular risk in this category of workers. It is therefore necessary to check that blood pressure values are always well controlled by drug therapy in all altitude conditions, and also to verify that these values do not show hyperkinetic adaptations (typical of hypertensive states) (Winkler et al., 2017) during the period of working at high altitude, thereby exposing the workers to an additional cardiovascular challenge, and hence to an increased cardiovascular risk. From the autonomic point of view the HR increase due to HA exposure seems to be due, similarly in both groups, to the rapid deactivation of the parasympathetic system, as demonstrated by the reduction in *baseline* values of the vagal-related indices of HRV (RMSSD and log HF), observed in both HT and NT individuals. Given that in non-treated hypertension the parasympathetic HRV indices are usually lower than those of normotensive individuals (Singh et al., 1998), our data suggest that the pharmacologic intervention in our HT individuals has somehow restored the normal autonomic modulation of resting HR. This is in line with what reported in many papers, and in particular with what has been reported as the main effect of ARB and ACE inhibitor treatments in hypertensive patients (Maciorowska et al., 2020). Interestingly, however, in our study the total power of the tachogram spectrum was still significantly reduced in baseline condition at SL in HT versus NT individuals. Usually, the total power of RR spectrum (i.e., the total variance of the RR signal) is calculated over a wide window of time, to allow capturing the RR oscillations also at the so called very-low and ultra-low frequencies, which are dependent on different mechanisms, ranging from the metabolic and thermoregulatory oscillations till the circadian rhythms. In our case, however, on 10 min HR recording the main part of total spectral power contains the low frequency oscillations of HR (around 0.1 Hz). These oscillations depend mainly on the functioning of the arterial baroreflex. It is therefore likely that a reduced sensitivity of the arterial baroreflex is still present in the group of HT subjects at rest, despite the drug treatment. As indirect proof of this hypothesis, during the phases of increased sympathetic activation related to the pre- and post-exercise phases, where the arterial baroreflex must be slightly inhibited to allow the simultaneous rise in blood pressure and HR, the TPs are reduced to similar values in the two groups of HT and NT. However, the analysis of the efficiency of the baroreflex mechanisms and their changes with HA was beyond the scope of this study and should have been evaluated by means of combined blood pressure and heart rate variability analysis. This aspect therefore remains an issue where additional investigating in future studies is needed.

Finally, looking at the EDR data, we found an interesting result: both factors, time and altitude exerted a significant effect, showing increased EDR at HA for both groups. However, the interaction altitude x group displayed a different behavior between HT and NT: HT showed significantly higher EDR already at baseline, in the morning upon awakening, compared to the NT group. Furthermore, HT showed higher rates of respiration at HA in comparison with NT and a larger difference with respect to SL at each of the other timepoints. Therefore, while NT individuals incremented in similar manner the estimated respiratory frequency in response to HA, HT individuals did not, and increased the respiratory frequency in all conditions in response to HA exposure. This suggests different mechanisms of adaptation of the lung to HA, both at rest and in preparation and response to and in response to exercise. In this paper we did not measure the tidal volume adaptation to HA, so we cannot clarify whether the increase in EDR represents a response to a failure to increase the lung volumes for example, during exercise. Although the mechanism of the altered ventilatory rate response to HA remains to be clarified, this adaptation pattern might be important in the hypertensive individuals, in whom right ventricular dysfunction even with a preserved function of the left ventricle has been reported (Vriz et al., 2018). This would predispose to the development of pulmonary hypertension, resulting in a possible altitude maladjustment in affected workers. This possibility needs to be further evaluated in future studies on acute adaptation to high altitude in hypertensive individuals.

We acknowledge that our study is limited because of its small sample size, due to the constraints of data collection at HA in workers during their official shifts. Therefore, our study should be considered as a pilot investigation aimed at exploring physical performance in workers exposed to CIH through 6MWT and HRV. On the other hand, we must mention that the workers carried out the 6MWT at the end of their working day, which could affect their performance, however, this could not be improved as these are the specific working conditions, thus it was done to the best of our ability. Another limitation of our study is that in the analysis of cardiac autonomic control, the data sets available were limited to short ECG recordings. It will therefore be necessary to extend the duration of these recordings up to 24 h (with particular attention to night hours) if more detailed evaluations of cardiac autonomic modulation in conditions of HA exposure are to be carried out.

## Conclusion

HT and NT miners exposed to CIH showed a comparable acute response of hemodynamic parameters, which might be due to the protective effect in HT of an effective antihypertensive treatment. However, HT individuals showed a larger effect of HA on HRV, as well different fast HR recovery (typically modulated by the parasympathetic system reactivation) after submaximal exercise.

Submaximal exercise performance measured by 6MWT did not significantly differ between SL and HA in both groups, despite a slight decrease of the covered distance at HA in comparison with SL appeared in both groups. However, HT independently from altitude, showed a significantly reduced performance with respect to NT. This information is clinically relevant since many workers are hypertensive and must perform physical work under these conditions.

Finally, exposure to HA showed a different adaptation of respiratory rate between NT and HT in all the conditions observed, which deserves further evaluation.

## Data Availability

The raw data supporting the conclusion of this article will be made available, upon request, by the authors, without undue reservation.
